# Levels and values of lipoprotein-associated phospholipase A_2_, galectin-3, RhoA/ROCK, and endothelial progenitor cells in critical limb ischemia: pharmaco-therapeutic role of cilostazol and clopidogrel combination therapy

**DOI:** 10.1186/1479-5876-12-101

**Published:** 2014-04-17

**Authors:** Jiunn-Jye Sheu, Pao-Yuan Lin, Pei-Hsun Sung, Yi-Ching Chen, Steve Leu, Yung-Lung Chen, Tzu-Hsien Tsai, Han-Tan Chai, Sarah Chua, Hsueh-Wen Chang, Sheng-Ying Chung, Chih-Hung Chen, Sheung-Fat Ko, Hon-Kan Yip

**Affiliations:** 1 Division of Thoracic and Cardiovascular Surgery, Kaohsiung Chang Gung Memorial Hospital and Chang Gung University College of Medicine, Kaohsiung, Taiwan; 2 Division of Plastic and Reconstructive Surgery, Department of Surgery, Kaohsiung Chang Gung Memorial Hospital and Chang Gung University College of Medicine, Kaohsiung, Taiwan; 3 Division of Cardiology, Department of Internal Medicine, Kaohsiung Chang Gung Memorial Hospital and Chang Gung University College of Medicine, Kaohsiung, Taiwan; 4 Center for Translational Research in Biomedical Sciences, Department of Biological Sciences, Kaohsiung Chang Gung Memorial Hospital, Kaohsiung, Taiwan; 5 Department of Biological Sciences, National Sun Yat-Sen University, Kaohsiung, Taiwan; 6 Divisions of General Medicine, Kaohsiung Chang Gung Memorial Hospital, Kaohsiung, Taiwan; 7 Department of Radiology, Division of General Radiology, Kaohsiung Chang Gung Memorial Hospital and Chang Gung University College of Medicine, Kaohsiung, Taiwan; 8 Institute of Shock Wave Medicine and Tissue Engineering, Kaohsiung Chang Gung Memorial Hospital, Kaohsiung, Taiwan

**Keywords:** Critical limb ischemia, Endothelial progenitor cells, Inflammation, Clopidogrel, Cilostazol therapy

## Abstract

**Objective:**

We tested the hypothesis that clopidogrel and cilostazol combination therapy could effectively attenuate systemic inflammatory reaction, facilitate proliferation of circulating endothelial progenitor cell (EPC), and improve the clinical outcomes of critical limb ischemia (CLI) in patients unsuitable for surgical revascularization or percutaneous transluminal angioplasty (PTA).

**Methods:**

A total 55 patients (mean age, 72 years; 56% female) were consecutively enrolled. Clopidogrel and cilostazol combination therapy was administered throughout the study period.

**Results:**

As compared with the baseline, circulating endothelial progenitor cell level (as shown by flow cytometry) was significantly increased (p < 0.003), whereas the CLI-related ulcers and painfulness were significantly improved (all p < 0.01) by day 90 after treatment. On the other hand, after clopidogrel and cilostazol combination therapy, galectin-3 level, lipoprotein-associated phospholipase A_2_ gene expression, and RhoA/ROCK-related protein expression in peripheral blood mononuclear cells were significantly suppressed (all p < 0.01). Eventually, by day 90, 5 patients (9.1%) died of other etiologies, 3 (5.5%) withdrew from the study, 6 (10.9%) required amputation, and the remaining 41 had satisfactory clinical improvement with complete wound healing in 9 (16.4%) patients.

**Conclusion:**

The results of the present study highlight that clopidogrel and cilostazol combination therapy may be considered to be an alternative method for treating patients with CLI unsuitable for surgical revascularization or PTA.

## Introduction

Atherosclerosis is a progressive and complex inflammatory process affecting both regional and systemic arteries [1-5]. Peripheral artery disease (PAD) caused by atherosclerotic lesions is an important manifestation of systemic atherosclerosis [6]. Patients with PAD may develop critical limb ischemia (CLI) at the late stage of the disease [7,8]. Treatment for CLI remains a tough challenge to clinicians. Without appropriate treatment, the 1-year mortality rate can be as high as 25% [9]. Failure in salvaging CLI can lead to major limb loss and high cost of patient care following amputation. Although surgical or endovascular revascularization have been used for the treatment of CLI with acceptable successful rate, for the patients who are not good candidates for surgical or endovascular intervention and those with failure of revascularization or bypass occlusion, the clinical outcomes remain dismal. Therefore, an alternative strategy for the treatment of such CLI patients is necessary.

Platelet activation and inflammation have been reported to play essential roles in the development of arterial atherosclerotic-obstructive syndrome (AAOS) [1-5,10-13]. Various biomarkers have been used for assessing platelet activity and inflammation in different clinical settings. Lipoprotein-associated phospholipase A_2_ (Lp-PLA_2_), also known as platelet-activating factor acetylhydrolase, is useful for predicting unfavorable outcome in patients after acute ischemic stroke [14-16]. Galectin-3, a biomarker of inflammatory response, is a useful predictor of prognostic clinical outcome in patients after acute myocardial infarction (AMI) [17-22]. Rho, a small monomeric GTPase, and Rho-associated kinases (ROCK), the immediate downstream targets of RhoA, are useful for monitoring sustained vasoconstriction, vascular remodeling, and inflammatory response in arterial obstructive diseases [23-27] as well as down-regulation and inhibition of endothelial nitric oxide synthase (eNOS) [28,29].

Clopidogrel, an adenosine diphosphatase (ADP) inhibitor, is currently utilized in acute arterial occlusive syndrome [10,30], after coronary artery stenting for inhibiting platelet activity and in-stent thrombus formation [30], and in secondary prevention and at high-risk prevention for atherothrombotic events [31,32]. Cilostazol, a phosphodiesterase III inhibitor for treating intermittent claudication owing to its pleotropic effects in reducing smooth muscle proliferation, limiting intimal hyperplasia after endothelial injury, inhibiting platelet activation and thrombus formation, and heightening anti-inflammation [33-35]. The purpose of this study was to test the hypothesis that clopidogrel and cilostazol combination therapy could effectively attenuate systemic inflammatory reaction through inhibiting Lp-PLA2 activity, galectin-3 and Rho/ROCK, facilitate mobilization of circulating endothelial progenitor cell (EPC) to circulation, and improve the clinical outcomes of CLI patients unsuitable for surgical revascularization or percutaneous transluminal angioplasty (PTA).

## Materials and methods

### Patients

Between September 2010 and October 2012, a total 55 CLI patients who fulfilled the following criteria were enrolled. Inclusion criteria of the patients included: (a) presence of Fontaine stage III-IV CLI presented with ischemic rest pain and ischemic skin lesions, either ulcers or gangrene; (b) featuring a reduced ankle-brachial index (ABI) less than 0.9 at rest and ankle systolic pressure less than 50 mmHg; (c) harboring diffuse long segmental infrapopliteal severe stenoses or occlusion and marked narrowing or lack of pedal arteries so that the patients were determined as not appropriate for surgical or endovascular interventions. The enrolled candidates were then managed with clopidogrel (75 mg/day) and cilostazol (50 mg twice per day) combination therapy. Patients who had allergy to the drug or hematologic disorder or bleeding/hemostatic problem and refused the treatment were excluded from the study. The Institutional Review Committee on Human Research at our institution approved this study protocol (the IBR number: 99-1688B) and informed consent was obtained from each study subject.

### Definition of ulceration wound and grade on healing

The wound classification (i.e., including wound size and wound grade on healing) in the present study was according to the Curative Health Services (CHS) wound grade scale [36] which was described as the followings:

Wound grade 1: defined as partial thickness involving only dermis and epidermis

Wound grade 2: defined as full thickness and subcutaneous tissues

Wound grade 3: defined as grade 2 plus exposed tendons, ligament, and/or joint

Wound grade 4: defined as grade 3 plus abscess and/or osteomyelitis

Wound grade 5: defined as grade 3 plus necrotic tissue in wound

Wound grade 6: defined as grade 3 plus gangrene in the wound and surrounding tissue

### Blood sampling

Blood samples for the assessments of circulating galectin-3 level and the Rho/ROCK activity and Lp-PLA2 gene expression in peripheral-blood mononuclear cells (PBMNCs) were collected via the antecubital vein prior to and at 90 day after the drug therapy.

### Measurement of galectin-3 level

After centrifugation, the aliquot of the samples was stored at -80°C before the assay for galectin-3 level. White blood cell (WBC) counts, biochemical measurements and electrolyte levels were performed with standard laboratory methods. Serum galectin-3 level was measured by duplicated determination with a commercially available ELISA method (R & D). The intra-observer variability of the measurements of galectin-3 levels was also assessed and the mean intra-assay coefficients of variance were all < 4.6%.

### Protocol for RNA extraction and reverse transcription qPCR analysis for relative mRNA expression of Lp-PLA_2_ of PBMNCs to β actin

The procedure and protocol for RNA extraction reverse transcription qPCR analysis were according to our previous report [16]. In details, the lysis/binding buffer (400 μL) (High Pure RNA Tissue Kit, Roche, Germany) and an appropriate amount of frozen PBMNCs were added to a nuclease-free 1.5 mL microcentrifuge tube, followed by disruption and homogenization of BPMNCs by using a rotor-stator homogenizer (Roche). The lysate in the microcentrifuge tube was then centrifuged for two minutes at 13,000 *g*. Only the superficially collected supernatant was utilized for subsequent steps. Absolute ethanol (200 μL) was then added to the lysate supernatant and mixed well. The entire sample in the upper reservoir was pipetted into a High Pure Filter Tube (Roche) that was placed in the Collection Tube (Roche). This sample was then centrifuged for 30 seconds at 13,000 *g* in a standard tabletop microcentrifuge. After that, the Filter Tube was removed from the Collection Tube and the flowthrough liquid was discarded. For each isolation, 90 μL of DNase incubation buffer was pipetted into a sterile 1.5 mL reaction tube, 10 μL of DNase I working solution was then added, mixed and incubated for 15 minutes at 25°C. Wash buffer I (500 μL) was then added to the upper reservoir of the filter tube, which was then centrifuged for 15 seconds at 8,000 *g*. The filter tube was removed from the Collection Tube and the flowthrough liquid was then discarded. Wash Buffer II (500 μL) was added to the upper reservoir of the Filter Tube, which was then centrifuged for 15 seconds at 8,000 *g* and the flowthrough was discarded. Wash buffer II (300 μL) was added to the upper reservoir of the filter tube, which was centrifuged for 2 minutes full-speed at approximately 13,000 *g*. The column was then carefully removed from the collection tube such that the column did not contact the flow through to avoid ethanol carryover. The filter tube was then inserted into a 1.5 mL nuclease-free and sterilized microcentrifuge tube. Elution Buffer (100 μL) was added to the upper reservoir of the filter tube; the tube assembly was then centrifuged for 1 minute at 8,000 *g* resulting in eluted RNA in the microcentrifuge tube.

Quantitative reverse transcription polymerase chain reaction (RT-qPCR) was conducted using LightCycler TaqMan Master (Roche, Germany) in a single capillary tube according to the manufacturer’s guidelines for individual component concentrations. The Lp-PLA_2_ forward (TGGCTTACCTTAGAACCCTGA) and reverse (TTTTGCTCTTTGCCGTACCT) primers were each designed based on individual exons of the target gene sequence to avoid amplifying genomic DNA.

During PCR, the probe was hybridized to its complementary single-strand DNA sequence within the PCR target. As amplification occurred, the probe was degraded due to the exonuclease activity of Taq DNA polymerase, thereby separating the quencher from reporter dye during extension. During the entire amplification cycle, light emission increased exponentially. A positive result was determined by identifying the threshold cycle value at which reporter dye emission appeared above the background.

### Western lot analysis of PBMNC specimens for Rho/ROCK activity

Equal amounts of extracted proteins from BPMNCs in each patient were loaded and separated by SDS-PAGE using 7% or 12% acrylamide gradients. The membranes were incubated with rabbit polyclonal antibodies against myosin phosphatase (MYPT), p-MYPT, myosin light chain, p-MLC, and small GTP-binding proteins: (1) RhoA, (2) Rac.

Proteins were transferred to nitrocellulose membranes which were then incubated in the primary antibody solution (anti-DNP 1:150) for two hours, followed by incubation with the second antibody solution (1:300) for one hour at room temperature. The washing procedure was repeated eight times within 40 minutes.

Immunoreactive bands were visualized by enhanced chemiluminescence (ECL; Amersham Biosciences), which was then exposed to Biomax L film (Kodak). For quantification, ECL signals were digitized using Labwork software (UVP). For oxyblot protein analysis, a standard control was loaded on each gel.

### Flow cytometric analysis for circulating endothelial cells (EPCs)

A flow cytometic method for identification of EPCs derived from peripheral blood has been reported in our recent studies and also those by others [37]. Briefly, the isolated MNCs (4 × 10^5^) were incubated for 30 minutes at 4°C in a dark room with monoclonal antibodies against kinase insert domain-conjugating receptor (KDR) (Sigma), the fluorescein isothiocyanate (FITC)-conjugated CD34 and the phycoerythrin (PE)-conjugated CD31, and CXCR4 to determine the EPC surface markers of CD31/CD34, CXCR4/CD34, and KDR/CD34. The control ligand (IgG-PE conjugate) was used to detect any nonspecific association and define a threshold for glycoprotein binding. For analysis of KDR, the MNCs were further incubated with PE-conjugated anti-mouse antibody made in goat. After staining, the MNCs were fixed in 1% of paraformaldehyde. Quantitative two-colored flow cytometric analysis was performed using a fluorescence-activated cell sorter (FACSCalibur™ system; Beckmen). Each analysis included 300,000 cells per sample. The assays for EPCs (E_1–3_) in each sample were performed in duplicate, with the mean level reported.

For the accuracy of flow cytometry, we had performed both isotype control and fluorescence minus one (FMO) control for each sample of flow cytometric examination. The results showed that only none or lesser than 0.1% of fluorescence spillover in each FMO control test.

Intra-assay variability based on repeated measurement of the same blood sample was low with a mean coefficient of variance being 3.9% and 3.6% in the patients and in normal subjects, respectively [35].

### Image studies

Chest radiographs, duplex scanning for assessing the arterial flow of lower extremity, 12-lead electrocardiogram, echocardiography, at least one time of magnetic resonance angiography image (MRA) or digital subtraction angiography of the lower extremities were performed upon hospitalization or at out patient department for evaluating the severity of obstructive arteries in the lower extremity.

### Medications

In addition to cilostazol and clopidogrel combination therapy, other commonly used drugs including statin, angiotensin converting enzyme inhibitors, calcium channel blocking agents, and isordil/vasodilatation agents were applied as needed by individual.

### Data collection and clinical follow-up

Detailed in-hospital and follow-up data at out patient department including age, gender, coronary risk factors, serum creatinine level and other related laboratory findings, adverse clinical events during study period and mortality were obtained.

### Statistical analysis

Continuous variables with normal distribution were expressed as mean ± SD. Categorical data were analyzed by Chi-square test and continuous variables were analyzed using paired *t* test. Statistical analysis was performed using SPSS statistical software for Windows version 13 (SPSS for Windows, version 13; SPSS Inc., IL, U.S.A.). A p-value of < 0.05 was considered statistically significant.

## Results

### Baseline characteristics of 55 study patients

**Figure 1:**
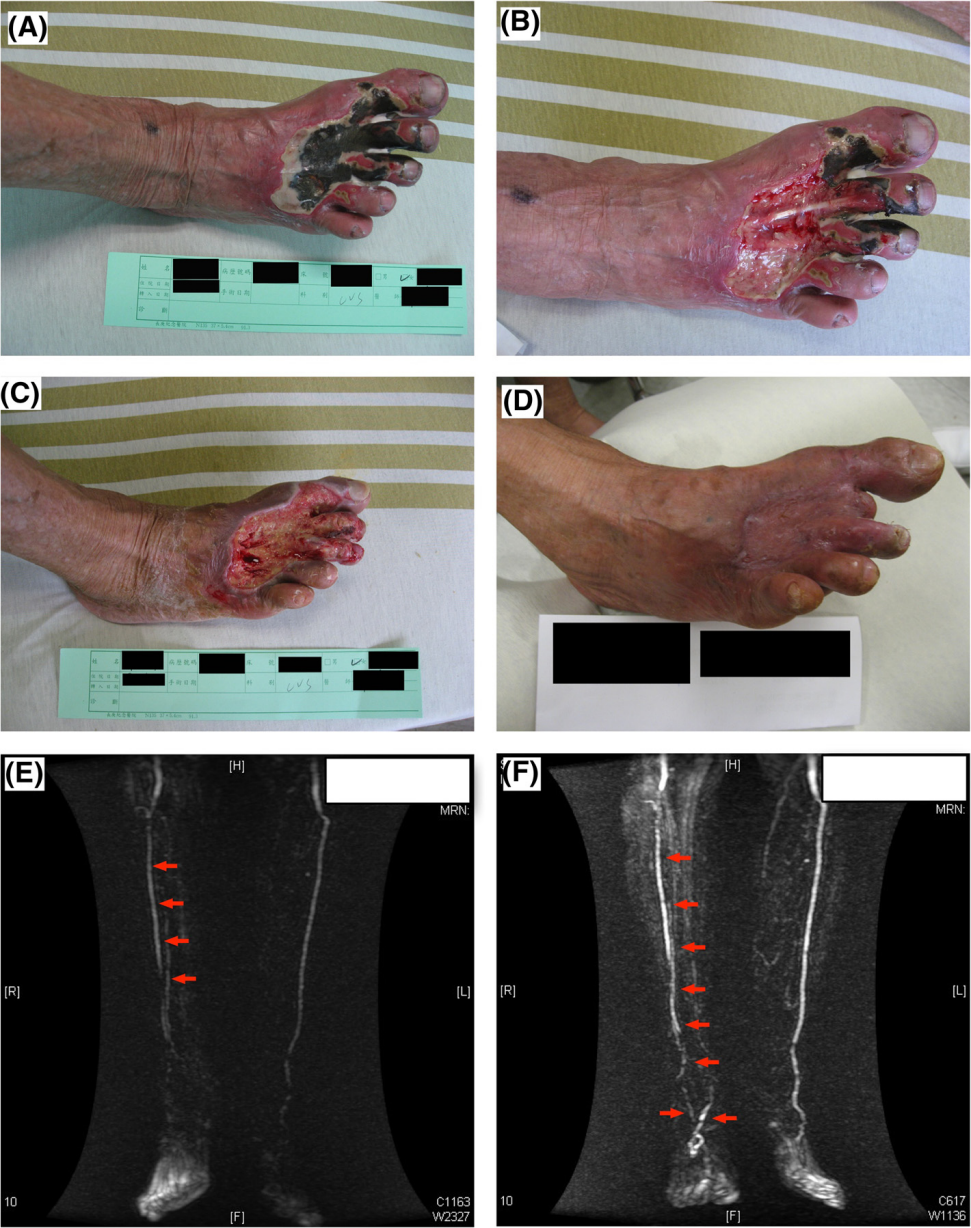
**Illustrating the time course of the critical limb ischemia (CLI) in one patient. A)** Indicated the wound appearance prior to treatment. **B)** Illustrated the wound situation by one week after debridement and combined therapy with clopidogrel and cilostazol treatment. **C)** Indicated the wound situation at one month after the treatment. **D)** Indicated wound situation by day 90 after the treatment. **E)** The result of magnetic resonance angiography image (MRA) prior to the combined therapy with clopidogrel and cilostazol showed the poor blood flow and total occlusion of arteries at the level just above the right ankle joint (red arrows). **F)** By day 90 after the combination therapy, the results of MRA showed that the new angiogenesis/small vessel density and collateral circulations were found to be present (red arrows).

The clinical data of the patients are summarized in Table 1. Most of the patients were of old age (mean age 72 years) and there was no gender predominance (56% female). Co-morbidities included hypertension (80%), diabetes mellitus (65%) and dyslipidemia (42%) while 27% patients had history of old stroke. In addition, 54% had varies degrees of chronic kidney disease including 27% were at end-stage. More than 50% had received statin therapy. Up to 18.2% of the patients had history of below knee or toe amputation. More than 80% of the patients suffered from lower leg or foot ulcers. Among them, 60% had ≥ grade 4 ulcer (Figure 1) combined with abscesses or osteomyelitis indicating that most of our patients endured severe ischemic complications.

**Table 1:** Baseline characteristics of 55 study patients

**Variables**	**% (n) or mean ± SD**
Age (yrs)	72 ± 11
Female gender	56.4% (31)
Diabetes mellitus	65.5% (36)
Hypertension	80.0% (44)
Current smoking	34.6% (19)
Dyslipidemia	41.8% (23)
Old stroke	27.3% (15)
Chronic kidney disease (stage I-IV)	27.3% (15)
End-stage renal disease on regular hemodialysis	27.3% (15)
Statin therapy*	50.9% (28)
ACEI/ARB therapy*	58.2% (32)
History of one leg amputation	18.2% (10)
Wound ulceration†	
Grade 0	18.2% (10)
Grade 1	3.6% (2)
Grade 2	5.5% (3)
Grade 3	14.6 (8)
Group 4	60.0% (32)

Data are expressed as mean ± SD or% (n).

*Indicate the drug had already given to the patients ≥ 1 month.

ACEI/ARB = angiotensin converting enzyme inhibitor/angiotensin II receptor type I blocker.

### Compared the changes of laboratory findings, circulating EPC and galectin-3 level, and Lp-PLA_2_ mRNA expression between day 0 and day 90 after clopidogrel and cilostazol combination therapy among 55 study patients

The laboratory findings are shown in Table 2. The red blood cell, white blood cell and platelet counts, and hemoglobin did not differ between day 0 and day 90 after clopidogrel and cilostazol combination therapy among the 55 study patients. However, as compared with day 0, the total cholesterol and low-density lipoprotein levels were significantly reduced, whereas the high-density lipoprotein was significantly increased at day 90 after clopidogrel and cilostazol combination therapy.

**Table 2:** Comparison of laboratory findings, circulating galectin-3 level and Lp-PLA2 gene expression between day 0 and day 90 after combined clopidogrel and cilostazol treatment among 55 study patients

**Variable**	**Day 0**	**Day 90**	**p-value**
Red blood cell count (×10^6^)	4.0 ± 0.8	3.8 ± 0.7	0.682
White blood cell count (×10^3^)	8.2 ± 3.2	8.0 ± 2.2	0.574
Platelet count (×10^3^)	250 ± 78	236 ± 115	0.483
Hemoglobin	11.5 ± 2.1	11.1 ± 2.1	0.811
Total cholesterol level	181 ± 52	152 ± 22	< 0.001
Low-density lipoprotein	106 ± 43	79 ± 24	< 0.001
High-density lipoprotein	55 ± 14	58 ± 14	0.001
Creatinine level (mg/dL)	3.23 ± 3.31	3.35 ± 3.46	0.731
Ac sugar	158 ± 76	148 ± 41	0.103
HbA1c	7.28 ± 1.65	6.80 ± 0.92	0.001
Lp-PLA2	1.46 ± 0.10	1.37 ± 0.09	< 0.001
Galectin-3 (ng/ml)	23.09 ± 4.17	15.53 ± 4.33	< 0.001
CD31+/CD34+ (%)	0.77 ± 0.48	0.80 ± 0.56	0.001
KDR+/CD34+ (%)	0.84 ± 0.55	0.89 ± 0.39	< 0.001
CXCR4+/CD34+ (%)	1.10 ± 0.70	1.11 ± 0.89	0.002

Data are expressed as mean ± SD.

Lp-PLA2 = lipoprotein-associated phospholipase A_2_.

The Ac sugar and creatinine level did not differ between day 0 and day 90 among these patients. However, as compared with day 0, the HbA1c levels were significantly reduced by day 90 after the combination therapy with clopidogrel and cilostatzol. Additionally, as compared with baseline, the circulating galectin level and the mRNA expression of Lp-PLA_2_, two indices of inflammatory biomarkers, were significantly lower by day 90 after clopidogrel and cilostazol combination therapy.

To elucidate whether the circulating numbers of EPCs (i.e., CD31+/CD34+, KDR+/CD34+, CXCR4+/CD34+) would be increased after clopidogrel and cilostazol combination therapy and statin, these circulating EPCs were measured using flow cytometry. As expected, these three surface EPC markers were significantly higher at day 90 than after these drug therapy than at day 0 prior to the treatment.

### Compared the protein expressions of RhoA/ROCK-related signaling between baseline and day 90 after the treatment

**Figure 2:**
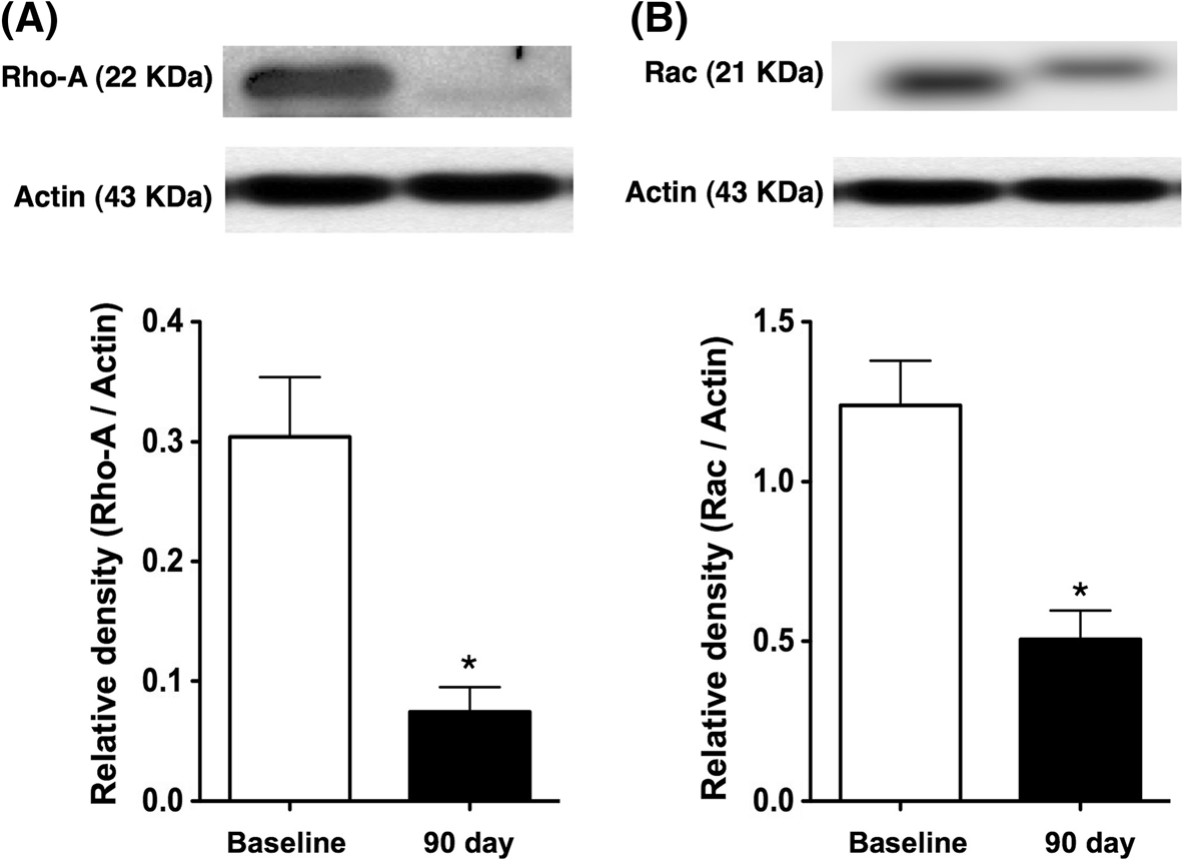
**The protein expression of Rho-A and Rac in peripheral leukocyte. A)** As compared with the baseline, the protein expression of Rho-A in peripheral leukocytes was significantly reduced in day-90 after clopidogrel and cilostazol combination therapy. * vs. baseline, p < 0.001. **B)** As compared with the baseline, the protein expression of Rac in peripheral leukocytes was significantly reduced in day-90 after clopidogrel and cilostazol combination therapy. * vs. baseline, p < 0.01.

**Figure 3:**
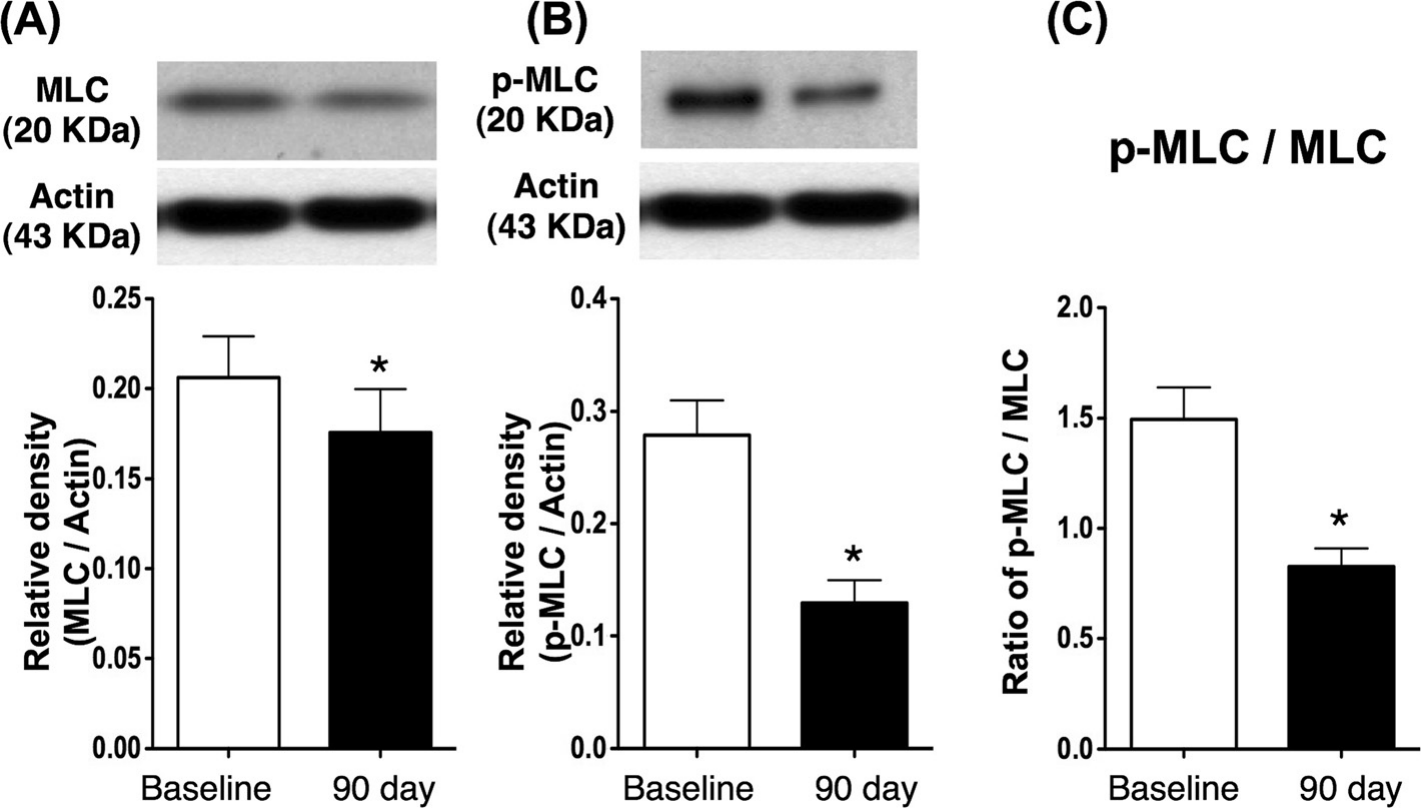
**The protein expression of MLC in peripheral leukocytes. A)** The total protein expression of myosin light chain (MLC) was significantly lower at day 90 after combination therapy than at baseline. * vs. baseline, p < 0.05.**B)** The protein expression of phosphorylated myosin light chain (p-MLC) was significantly lower at day 90 after combination therapy than at baseline. * vs. baseline, p < 0.001. **C)** As compared with baseline, the ratio of p-MLC to total MLC was significantly lower by day 90 after the combination therapy. * vs. baseline, p < 0.01.

**Figure 4:**
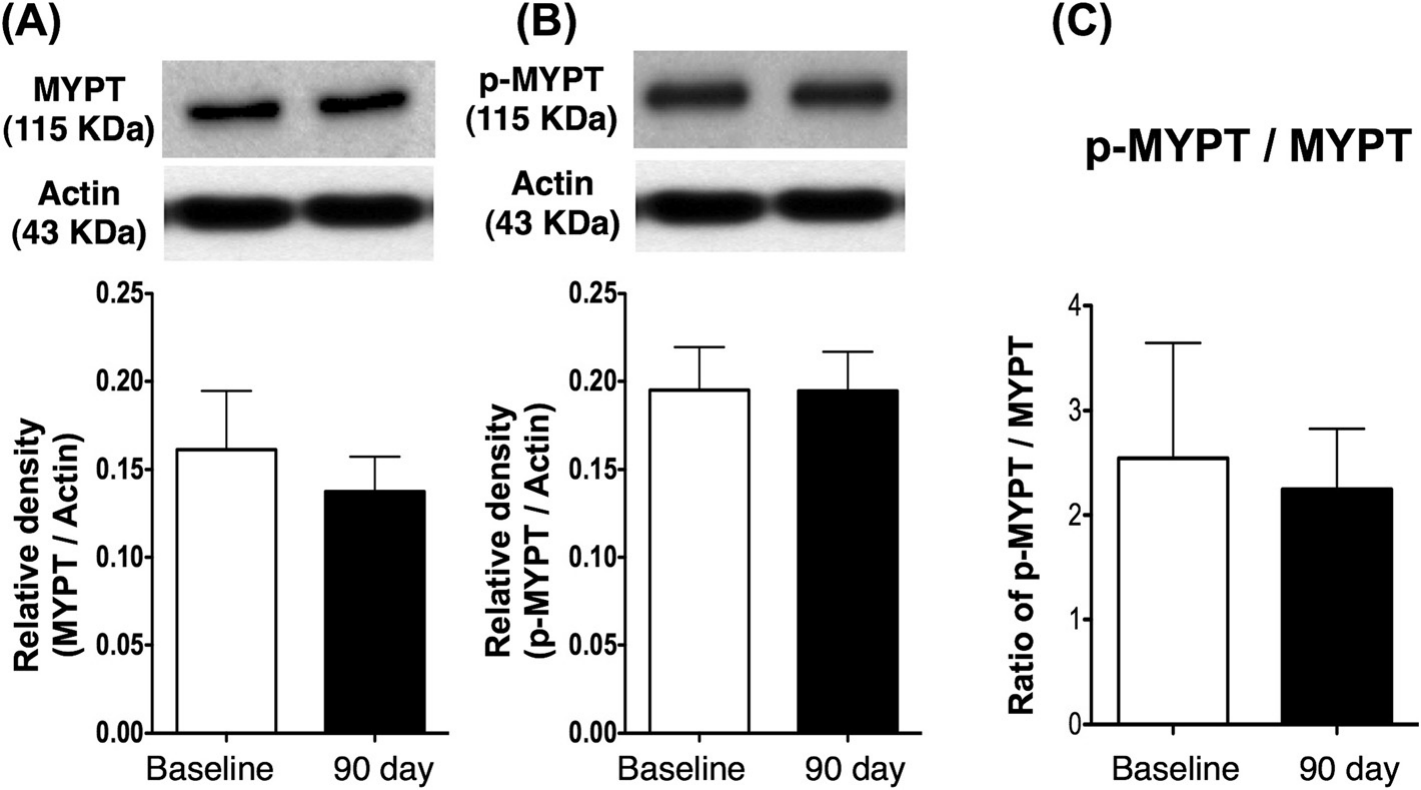
**The protein expression of MYPT in peripheral leukocytes.** The protein expressions of total myosin phosphatase (MYPT) **(A)**, phosphorylated (p)-MYPT **(B)** and the ratio of total MYPT to p-MYPT **(C)** did not differ between baseline and at day 90 after the treatment, p > 0.05.

The protein expressions of RhoA and Rac, two small GTP-binding proteins, were significantly lower in day 90 as compared to the baseline (Figure 2). Additionally, the protein expressions of total MLC, p-MLC and the ratio of p-MLC to total MLC were significantly lower in day 90 than prior to the treatment (Figure 3). These finding implicated the ROCK activity was notably reduced on day 90 after clopidogrel and cilostazol combination therapy. However, the protein expressions of total MYPT, p-MYPT the ratio of p-MYPT to total MYPT did not differ between the baseline and day 90 (Figure 4).

### Clinical outcomes on day 90

The clinical outcomes of 55 patients are shown in Table 3. As compared with day 0, the grade of ulcerative wound was significantly reduced and subjective painful sensation due to the ulcerative wound was significantly improved on day 90. These findings implicate that clopidogrel and cilostazol combination therapy significantly improved the would healing process.

**Table 3:** 90-Day clinical outcomes among 55 study patients

**Variables**	**Day 0**	**Day 90**	**p-value**
Mean ulcerative wound (grading)	2.90 ± 1.60	2.11 ± 1.64	< 0.001
Painful sensation due to CLI	92.7% (51)	19.8% (17)	< 0.001
90-day individual clinical outcome			
Gastric ulcer with bleeding		1.8% (1)	
Status of CLI (90-day end point)			
Amputation due to worsening CLI		10.9% (6)	
Stationary*		34.6% (19)	
Greater improvement†		23.6% (13)	
Complete healing		16.4% (9)	
Withdraw		5.5% (3)	
Sepsis-cause death		5.5% (3)	
Cardiovascular-caused death		1.8% (1)	
Cancer-cause death		1.8% (1)	

Data expressed as mean ± SD or% (n).

*Defined as the ulcerative wound had more clean without progressive worsening.

†Defined as the ulcerative wound healing with > 50% of the area.

During the study period, one patient experienced gastric ulcer with bleeding and the requirement of blood transfusion. By day 90, 6 patients had to receive toe amputation due to worsening CLI. On the other hand, 19 patients had a stationary setting of CLI, 13 patients had greater improvement of CLI condition and 9 patients had complete healing of the CLI. Loss of follow-up was noted in three patients. Five patients died during the study period, including three with sepsis and shock, one with severe cardiovascular insult, and one with colon cancer.

## Discussion

This study, which investigated the impact of clopidogrel and cilostazol combination therapy on 90-day clinical outcome of 55 high-grade CLI patients unsuitable for surgical revascularization or PTA, yielded several striking implications. First, wound healing was remarkably improved, whereas the painfulness owing to ischemia-related ulcers was markedly reduced by day 90 after clopidogrel and cilostazol combination therapy. Second, after combination therapy, only few patients required the amputation treatment. Third, galectin-3 level, Lp-PLA_2_ gene expression, and RhoA/ROCK-related protein expression in PBMNCs, three indexes of inflammation, were substantially suppressed, whereas circulating EPC number was notably increased on day 90 after combination therapy. Accordingly, the results of the present study highlight that clopidogrel and cilostazol combination therapy may be considered as an alternative method for treating high-grade CLI patients with no surgical or endovascular revascularization.

A strong association between inflammation and atherosclerosis and acute arterial occlusive syndrome has been extensively investigated [1-5,10,13,16]. One recent study has shown that circulating level of galectin-3, an inflammatory biomarker, was significantly increased in patients after AMI and is useful for predicting untoward clinical outcome in AMI patients undergoing primary coronary intervention [22]. The present study also reveals marked elevation of galectin-3 level in patients with high-grade CLI suggesting that in addition to acute coronary arterial obstruction, galectin-3 can also be regarded as a biomarker for assessing chronic arterial ischemia. Of note, the circulating level of this biomarker was significantly suppressed at day 90 after clopidogrel and cilostazol combination therapy.

In the present study, in order to evaluate the role of WBC in the inflammatory process under the clinical setting of CLI, besides circulating level of galectin-3, the cellular level of inflammation in both mRNA expression (i.e., transcription level, i.e., Lp-PLA_2_) and protein express (translation level, i.e., RhoA/ROCK) were prospectively assessed. Interestingly, our recent study has displayed that Lp-PLA_2_ mRNA expression of PBMNC was significantly elevated in patients after acute ischemic stroke and elevated Lp-PLA_2_ mRNA expression can predict unfavorable outcome in such patients [16]. In addition to occlusion of the cerebral arteries [16], our results also show markedly elevated level of Lp-PLA_2_ mRNA expression in PBMNCs in CLI patients with peripheral arterial occlusion. In this way, our present finding strengthens that of our recent study [16]. Of particular importance was that this gene expression in PBMNCs was significantly attenuated by day 90 after clopidogrel and cilostazol combination therapy. Based on the present study, we postulate that galectin-3 and Lp-PLA_2_ mRNA may also be valuable biomarkers for assessing acute and chronic inflammatory changes.

A body of previous studies have shown that ROCK signaling pathway mediates in the sustained vasoconstriction, smooth muscle proliferation, vascular remodeling, hypertension and inflammatory reaction [23,27,38] and participates in down-regulation and inhibition of endothelial nitric oxide synthase (eNOS) [28,29]. Additionally, abundant evidences have revealed that peripheral leukocyte ROCK activity is an useful biomarker for predictive of co-morbidity of cardiovascular disease and long-term mortality in patients with cardiovascular disease [39,40]. In the present study, as compared with the baseline, our results reveal that the protein expressions of RhoA and Tac (two small GTP-binding proteins which mediates the pathological process of ROCK) were remarkably reversed whereas the total MLC and phosphorylated MLC (indicator of ROCK activity), and the ratio of phosphorylated MLC to total MLC were substantially attenuated on day 90 after clopidogrel and cilostazol combination therapy. These findings imply that combination therapy may have reduced the pro-inflammatory effect of WBCs despite no change in WBC counts between days 0 and 90 of study period (Table 2).

Clinical observational study have previously demonstrated that the 1-year mortality rate can be as high as 25% for CLI patients without appropriate treatment [9]. The most important finding in the present study was that besides marked suppression on inflammatory biomarkers, the clinical outcome of our patients with high-grade CLI without surgical or endovascular intervention was notably improved after clopidogrel and cilostazol combination therapy. Additionally, the blood sugar, HbA1c, total cholesterol level and LDL were also markedly reduced on day 90. Therefore, we suggest that the 90-day clinical improvement in CLI patients could be due to not only the effect of combination therapy but was also due to the well control of traditional CAD risk factors. The COURAGE Trial [41] has emphasized the clinical outcome of optimal medical therapy without percutaneous coronary intervention (PCI) in patients with stable coronary artery disease is similar to those underwent PCI on long-term follow-up. The results from Courage study [41] may support the findings of our study. Therefore, in view of the previous study outcomes [9,40], the results of the present study encourage the use of clopidogrel and cilostazol combination therapy for those CLI patients who are not the candidate for surgical or endovascular intervention.

The exact mechanisms of clopidogrel and cilostazol combination therapy in CLI remain unclear. We propose that three effects might be generated. First, marked clinical improvement with resumption of distal blood flow could be observed in our patients (Figure 1) indicating enhancement of vascularities in the lower extremities after treatment. Concurred with a recently published article with significantly enhanced angiogenesis and number of EPCs in the ischemic zone in a rat CLI model after combination therapy [42], remarkably elevation of the circulating level of EPCs was found in our patients after similar treatment for 90 days. Second, studies have previously reported that proliferation of neo-intimal growth of vascular smooth muscle cells (i.e., neointimal hyperplasia) plays a key role in the progression of restenosis and PAD as well as restenosis after PAD intervention [43-45]. Additionally, as described in many studies clopidogrel and cilostazol harbor anti-platelet and anti-thrombotic effects while cilostazol can inhibit smooth muscle cell proliferation [10,30-35]. According to the results of these studies [10,30-35,43-45], we believe that such effects may also contribute to the delightful outcome of among our patients. Third, as revealed by our results, clopidogrel and cilostazol combination therapy elicit potent anti-inflammatory effect which is helpful in limiting the exacerbation of ulcers and augmenting would healing.

**Figure 5:**
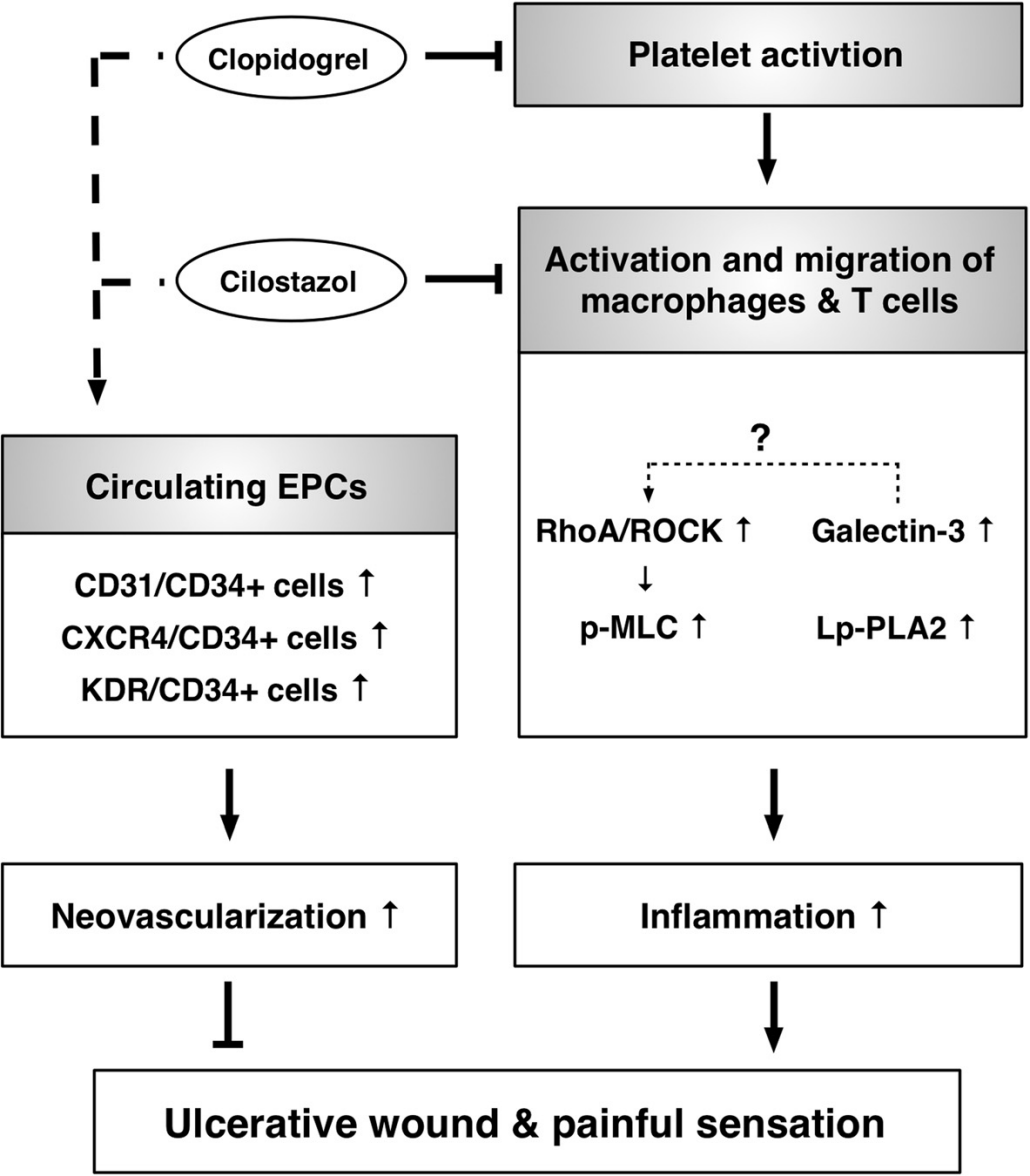
**Proposed mechanisms underlying the effects of combined clopidogrel and cilostazol therapy on improving outcomes of CLI based on the findings of the present study.** EPC = endothelial progenitor cells; ROCK = Rho-associated kinases; Lp-PLA_2_ = Lipoprotein-associated phospholipase A_2_; MLC = myosin light chain.

The proposed mechanisms of clopidogrel and cilostazol combination therapy in improving clinical outcome of CLI patients have been summarized in Figure 5 and described as the followings based on the findings of the current study and the previous reports [42,46,47]:

Platelet activation in setting of CLI stimulated the activation and migration of peripheral leukocytes (i.e., macrophages and T-cell) [46]. Both activated macrophages and T-cells then produced the galactin-3 which, in turn, promoted the focal adhesion turnover, cell migration, and RhoA activation [47]. Additionally, Lp-PLA2 enhanced the generation of inflammatory cytokines and up-regulated the inflammatory process. Finally, galactin-3, Lp-PLA2 and RhoA/ROCK synergistically promoted the inflammation and ultimately worsened the wound ulcerations and painful sensation (Figure 5). On the other hand, combine therapy with clopidogrel and cilostazol enhanced angiogenesis/neovascularization [42] which, in tern, improve blood flow in the ischemia zone, and finally, improved the wound healing.

### Study limitations

This study has limitations. First, the sample size of the present study was relatively small. However, the preliminary results clopidogrel and cilostazol combination therapy in CLI patients who are not amendable by surgical or endovascular methods are promising. Second, the 90-day study period in this initial trial was relatively short. The clinical benefits or drawbacks after long-term combination therapy are not known. Third, in addition to clopidogrel and cilostazol combination therapy, patients were also subjected to statin and ACI/ARB treatment during the study period and thus the effects or interaction of these drugs could hardly be validated. Fourth, although the methodology of EPC measurement in the present study was based on a validated method [35], a two-color assay with each of CD31, CXCR4 and KDR evaluated against CD34 might not accurately enumerate all of the EPCs. Finally, this study did not provide a placebo or control group. Therefore, we did not provide accurate information for how much additional benefit that the patients actually gained after receiving the combined treatment.

In conclusion, the results of the present study highlight that clopidogrel and cilostazol combination therapy may be considered as an alternative method for treating CLI patients who were not appropriate candidates for surgical revascularization or PTA.

## Competing interests

The authors declare that they have no competing interests.

## Authors' contributions

JJS and HKY designed the experiment and clinical treatment. PHS, YCC, SL, YLC, THT, HTC, SYC, CHC, and YHK were responsible for the laboratory assay and troubleshooting. HWC provide the statistical analysis. SC,CKS, SFK and HKY participated in refinement of experiment protocol and coordination and helped in drafting the manuscript. All authors have read and approved the final manuscript.
